# Laxative effects and mechanism of action of Brazilian green propolis

**DOI:** 10.1186/1472-6882-12-192

**Published:** 2012-10-22

**Authors:** Mamoru Kakino, Hiroshi Izuta, Kazuhiro Tsuruma, Yoko Araki, Masamitsu Shimazawa, Kenji Ichihara, Hideaki Hara

**Affiliations:** 1 Molecular Pharmacology, Department of Biofunctional Evaluation, Gifu Pharmaceutical University, 1-25-4 Daigaku-nishi, Gifu, 501-1196, Japan; 2 Nagaragawa Research Center, API Co., Ltd, 692-3 Nagarayamasaki, Gifu, 502-0071, Japan

**Keywords:** Propolis, Laxative, Acetylcholine receptor, Water extract

## Abstract

**Background:**

Brazilian green propolis is reported to have wide range of biological properties including antibacterial, anti-inflammatory, anti-influenza, and antioxidant activities. In the digestive system, a protective effect of propolis on gastric ulcer has been reported, but a laxative effect has not yet been reported. We investigated the effect and the mechanism of action of water and ethanol extracts of Brazilian green propolis.

**Methods:**

We examined the laxative effect of propolis on stool frequency by administering orally an ethanol extract of propolis (EEP) or a water extract of propolis (WEP) at 10, 50, 100, or 500 mg/kg to normal mice. We then investigated the effects of propolis using constipation model mice induced by two types of drugs, loperamide (a μ opioid receptor agonist) and clonidine (an α-2 adrenergic receptor agonist). We also investigated the effects of WEP on gastrointestinal transit and contractional tension of the ileum to uncover the mechanism of action of WEP.

**Results:**

Treatment with WEP, but not with EEP, significantly increased the weight of stools (p<0.01 at 500 mg/kg). WEP treatment significantly restored stool frequency and stool weight in clonidine-induced constipation model mice, but not in loperamide-induced constipation model mice. WEP treatment did not affect gastro-intestinal transit, but significantly increased the contractional tension of the isolated ileum of guinea pigs. This increase was inhibited by an acetylcholine receptor antagonist (atropine), but not by a 5-HT receptor antagonist (GR113808).

**Conclusion:**

These findings indicate that WEP has laxative effects both in normal mice and in clonidine-induced constipation model mice. The laxative effects of WEP might be mediated by increased contractional tension of the ileum exerted at least in part *via* activation of an acetylcholine receptor.

## Background

Propolis is a hard solid substance that honeybees make by mixing botanical materials or resins to protect their hive from adverse environmental factors such as bacteria, mites, rain, etc. The chemical constituents of propolis vary according to the production region, and four types of propolis are recognized worldwide: European, Brazilian, Cuban, and Taiwanese [1]. Brazilian propolis is further classified into 12 types [2]. The wide variation in chemical constituents of propolis reflects their botanical origins, and pharmacological reports of propolis fundamentally differ depending on botanical origin [3-7].

Brazilian “green” propolis is made when honeybees mix their own saliva and very small pieces of plant material that they gather from fresh tops of *Baccharis dracunculifolia*[8-11]. Brazilian green propolis contains various constituents and the ethanol extract of propolis (EEP) is reported to have a wide range of biological properties, such as anti-bacterial [12], anti-inflammatory [13], anti-hypertensive [14-16], anti-tumor [17], anti-hyperlipidemic [18], and antioxidant [19] activities.

Biological activity has also been reported for the water extract of propolis (WEP), including neuroprotective [20], anti-influenza [21-23], antioxidant [19], and anti-hyperglycemic [24] effects. In many reports, caffeoylquinic acids are recognized as the major active constituents [19,20,22-24]. The EEP and WEP had much stronger antioxidant activities against all types of reactive oxygen species (ROS) when compared to the activities of other bee products, such as royal jelly and bee pollen [25]. A comparison of EEP and WEP indicated similar efficacies for the scavenging of O_2_^.-^, but a ten-fold higher efficacy of WEP for scavenging H_2_O_2_ and OH^.^[21].

In general, compounds such as chlorogenic and ferulic acids can be detected in the water-soluble fraction of Brazilian green propolis, while compounds such as artepillin C (4-hydroxy-3,5-diprenylcinnamic acid), isosakuranetin, and drupanin (4-hydroxy-3-prenylcinnamic acid) can be detected in the ethanol-soluble fraction. On the other hand, *p*-coumaric acid, caffeoylquinic acids (3,5-dicaffeoylquinic acid, 3,4-dicaffeoylquinic acid, and 3,4,5-tricaffeoylquinic acid) can be detected in both propolis fractions [14].

Constipation is a symptom rather than a specific disease. It has many causes, including chemical compounds (e.g., morphine, clonidine, etc.), dietary habits (e.g., low-fiber diet, low-vitamin diet, high-fat diet, high-protein diet, etc.), composition of intestinal flora, pregnancy, and psychological stress [26]. Many types of purgative drugs have been identified, but most of these drugs induce severe side effects [27].

The effect of propolis on constipation has not been previously reported, but its use in traditional, not scientifically demonstrated, medicine indicates that it may have a laxative effect. Propolis can also have antibacterial effects that can interfere with the enteral environment and indirectly affect egestion. The purpose of this study was to investigate the potential laxative effect of propolis and to study the underlying mechanism using constipation model mice and various receptor antagonists.

## Methods

### Materials

Brazilian green propolis was supplied by API Co., Ltd. (Gifu, Japan). Loperamide hydrochloride, clonidine hydrochloride, and acetylcholine chloride were purchased from Wako Pure Chemical Co., Ltd. (Osaka, Japan). GR113808, a 5-HT_4_ receptor antagonist, was purchased from Sigma-Aldrich Corp., (St. Louis, MO, USA). Atropine sulfate monohydrate was purchased from Junsei Chemical Co., Ltd. (Tokyo, Japan). Serotonin hydrochloride (5-hydroxytryptamine hydrochloride) was purchased from Tokyo Kasei Kogyo Co. Ltd. (Tokyo, Japan).

### Extraction procedures of EEP and WEP

EEP: Brazilian green propolis (50 g) was fractured into pieces, added to 3.5 times its weight of 95% ethanol, gently mixed for 3 h, centrifuged at 400 g for 15 min, and the resulting extract was filtered through filter paper (5 μm pore size), stored at −20°C overnight, filtered again through filter paper (5 μm) to yield 12 g of EEP by evaporation.

WEP: Brazilian green propolis (50 g) was fractured into pieces, added to 5 times its weight of water, gently mixed for 4 h at 40-50°C, centrifuged at 1,000 g for 10 min, filtered through filter paper (5 μm), stored at 4°C overnight, filtered again through filter paper (5 μm) to yield 10 g of WEP by freeze dehydration. We examined with two different Lots of WEP and got almost the same result. A voucher specimen of propolis was deposited at the Nagaragawa Research Center, API Co., Ltd.

### Animals and ethical approval

Male ddY mice (5 weeks old, 27–29 g) and male Hartley guinea pigs (5 weeks old, 250-300 g) were purchased from Japan SLC (Hamamatsu, Japan). The animals were housed at a controlled room temperature (24.5-25.0°C) with a 12/12 h light/dark cycle. Food pellets [(CE-2 (for mice) or CG-7 (for guinea pigs), CREA Japan, Inc., Tokyo, Japan)] and tap water were provided *ad libitum*. The mice were acclimatized for one week before all experiments. All animal experiments were carried out according to the “*Principles of Laboratory Animal Care*” (NIH publication number 85–23, revised 1985) and “*Guidelines of the Animal Investigation Committee of Gifu Pharmaceutical University*.” All experiments were approved by the animal investigation committee of Gifu Pharmacological University.

### Stool parameters in normal mice

**Figure 1:**
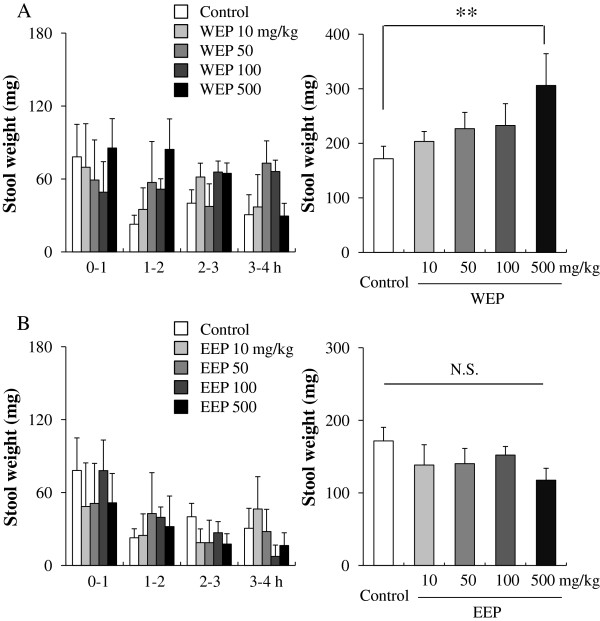
**Effects of orally administered propolis extracts on stool weight in normal mice.** (**A**): water extract of propolis (WEP); (**B**): ethanol extract of propolis (EEP). Data are shown as the means ± S.E.M., n=6, **p < 0.01 (one-way ANOVA by Dunnett’s multiple comparison tests), N.S.: not significant.

The mice were divided into nine groups (Control (n=6), WEP (four groups of different dosage, n=6 individually), and EEP (four groups of different dosage, n=6 individually)). WEP (10, 50, 100, and 500 mg/kg) and EEP (10, 50, 100, and 500 mg/kg) were orally administered (Figure 1A, B). WEP and EEP were suspended in 10% gum arabic at each concentration and administered at a volume of 0.1 ml/10 g body. The mice were food-deprived after the administration of WEP and EEP. The wet weights of stools from each mouse were measured at 1 h intervals for 4 h (e.g., 0–1 h, 1–2 h, 2–3 h, 3–4 h after administration).

### Induction of constipation and stool parameters in the two types of constipation mice

The mice were divided into three groups; control (n=6), vehicle (n=6), and WEP (n=6) in each experiment. The mice were administered WEP at 500 mg/kg and then administered clonidine hydrochloride (200 μg/kg) 45 min after or loperamide hydrochloride (5 mg/kg) 1 h after WEP administration. The frequency and weight of stools from each mouse were measured at 2 h intervals for 6 h (e.g., 0–2 h, 2–4 h, 4–6 h, etc.). Measurements were initiated 15 min after administration in loperamide-induced constipation model mice or immediately after administration of clonidine hydrochloride in clonidine-induced constipation model mice.

### Gastro-intestinal (GI) transit

The mice were divided into 2 groups (control (n=6) and WEP (n=6)). The mice were fasted for 14 h with water available *ad libitum* before the experiments. WEP was orally administered, then 30 min later, charcoal meal (5% charcoal/10% gum arabic) was administered orally (at a volume of 0.1 ml/10 g body). The mice were sacrificed 20 min later by cervical dislocation and the small intestine was carefully isolated from the pylorus. For each mouse, GI transit was calculated as the percentage of the distance traveled by the charcoal relative to the whole length of the small intestine. The GI transit (%) was calculated according to the equation below.

(1) $$\text{GI transit}\;\left(\%\right)=\left(\text{distance traveled by the charcoal}\right)/\left(\text{total length of the small intestine}\right)\times 100$$

### Contraction of isolated ileum by the Magnus method

The guinea pigs (n=3) were killed by cervical dislocation one by one. We can gain six isolated ileum tubes (1–1.5 cm) from each guinea pig because we can measure contractional tension of six isolated ileum tubes spontaneously with magnus system with six organ bathes. Segments of guinea pig intestine (ileum, latter half of the intestine) were suspended at a maximum tension of 1.0 g in automatic organ bath (Panlab Technology for Bioresearch, Barcelona, Spain) filled with 25 ml of Tyrode’s solution (137 mm NaCl, 5 mm KCl, 2.5 mm CaCl_2_-2H_2_O, 0.1 mm MgCl_2_-6H_2_O, 0.3 mm NaH_2_PO_4_-2H_2_O, 11.9 mm NaHCO_3_, and 5.6 mm glucose, pH 7.4). The minimum resting tension of the suspended intestine was determined as the basal tension. Spontaneous movement was monitored every 0.5 second with a recorder (Octal Bridge Amp; AD instruments, Castle Hill, Australia) *via* a transducer (PowerLab 8/30; AD Instruments). WEP solution was prepared in Tyrode’s solution at a concentration of 10 mg/ml. WEP solution was cumulatively added to the organ bath at 25, 75, 250, and 750 μl for final concentrations of 10, 40, 140, and 440 μg/ml, respectively (n=6). Atropine (an acetylcholine receptor antagonist) was prepared in physiological saline at concentration of 100 μg/ml and 250 μl was added to the organ bath for a final concentration of 1 μg/ml (n=4). A solution of GR113808 (a 5-HT_4_ receptor antagonist) was prepared in DMSO at a concentration of 1 mg/ml and 50 μl was added to the organ bath for a final concentration of 2 μg/ml (n=6). Atropine and GR113808 were added 10–15 min before WEP administration. Maximum tensions and average tensions were calculated; average tensions were determined with the monitored scores during 15 second and 5 min after administrations of the samples.

### Statistical analysis

Data are presented as mean ± S.E.M. Statistical comparisons were made with the Student’s *t*-test, the one-way ANOVA with Dunnett’s multiple comparison test or two-way repeated measure analysis of variance (ANOVA) with *t*-test; *p<0.05, **p<0.01 (JSTAT for Windows; Vector, Tokyo, Japan).

## Results

### Effects of WEP and EEP in normal mice

When WEP (10, 50, 100, and 500 mg/kg, n=6) was orally administered to normal mice, the wet weight of stools was significantly increased from 171.7 ± 18.7 to 306.0 ± 49.5 g at the 500 mg/kg administration dose (Figure 1A). Dose-dependent increases were seen at the lower concentrations (203.3 ± 18.3, 226.8 ± 29.9, and 232.7 ± 39.9 g at 10, 50, and 100 mg/kg, respectively). The frequency, reported as the numbers of stool beads, showed the same increasing trend, but the differences were not statistically significant (data not shown). Administration of EEP (10, 50, 100, and 500 mg/kg, n=6) had no effect on the stool weight (138.4 ± 27.9, 140.3 ± 20.9, 152.1 ± 11.9, and 117.6 ± 16.4 g at EEP doses of 10, 50, 100, and 500 mg/kg, respectively) (Figure 1B).

### The effect of WEP on stool parameters in two types of constipation model mice

**Figure 2:**
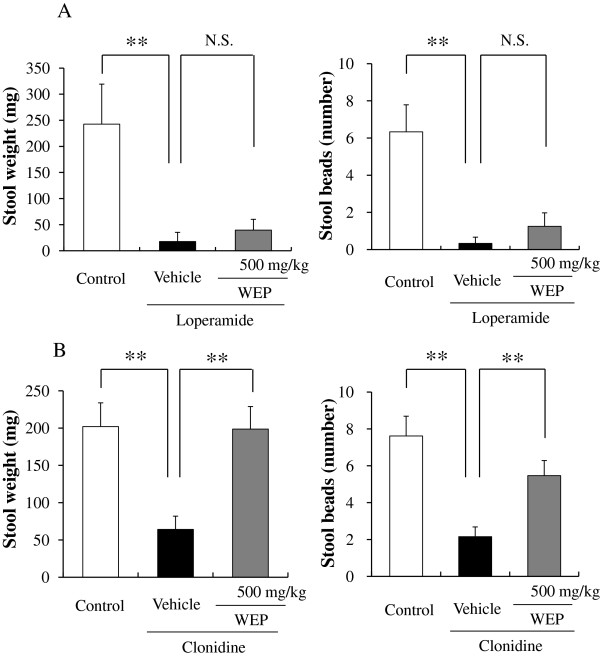
**Effects of an orally administered water extract of propolis (WEP) on stool weight and stool number.** (**A**): loperamide-induced constipation model mice; (**B**): clonidine-induced constipation model mice. Data are shown as the means ± S.E.M., n=3 to 13, **p < 0.01 (one-way ANOVA by Dunnett’s multiple comparison test), N.S.: not significant.

Constipation *via* μ-opioid receptor inhibition was induced by loperamide. Administration of loperamide significantly reduced the stool weight from 242.5 ± 76.8 (n=3) g to 39.6 ± 20.7 (n=3) g and stool frequency from 6.3 ± 1.5 (n=3) to 0.3 ± 0.3 (n=3). Administration of WEP showed a slight tendency to increase stool weight and stool frequency, but the differences were not statistically significant (Figure 2A).

Constipation *via* the α-2 adrenergic receptor was induced by clonidine. Administration of clonidine significantly reduced the stool weight from 201.2 ± 31.7 (n=13) g to 64.1 ± 17.8 g and stool frequency from 7.6 ± 1.1 (n=13) to 2.2 ± 0.5. WEP significantly increased the stool weight and stool frequency to 198.7 ± 30.2 (n=13) g and 5.5 ± 0.8 (n=13), respectively.

### Effect of WEP on GI transit

WEP at 500 mg/kg did not affect GI transit.

### Effect of WEP on the tension of isolated ileum of guinea pigs

**Figure 3:**
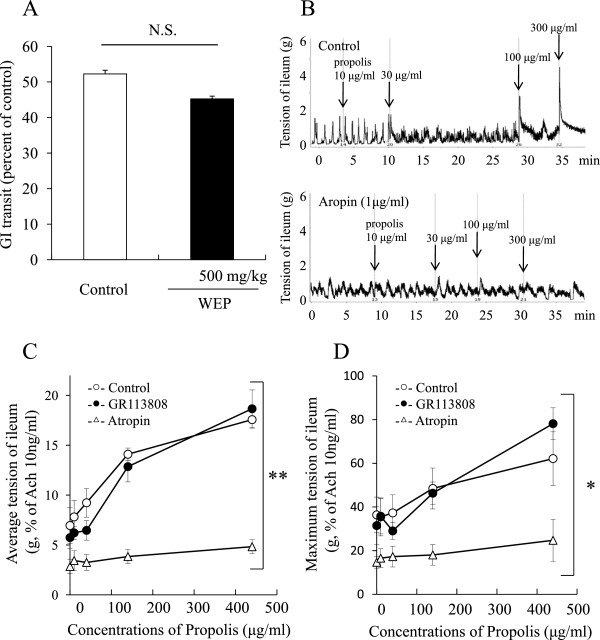
**Effect of an orally administered water extract of propolis (WEP) on GI transit and isolated guinea pig ileum.** (**A**): GI transit; (**B**): representative figure of ileum contraction without/with atropine; (**C**): effect of WEP on average tension Δ 5 min after the administration; and (**D**): on maximum tension of the ileum. Data are shown as the means ± S.E.M., n=6, *p < 0.05, **p < 0.01, (two-way repeated measure ANOVA), N.S.: not significant.

WEP caused dose-dependent increases in the ileum tension. Intestinal tension of the suspended ileum showed immediate but temporary increments in response to WEP, and tension remained slightly higher than the basal tension (Figure 3B).

We pretreated the suspended ileum with atropine, an acetylcholine receptor antagonist, and SR113808, a 5-HT_4_ receptor antagonist, before treatments with WEP (Figure 3C and D). Atropine, but not GR113808, significantly inhibited the incremental increases in maximum and average tension induced by administration of WEP.

## Discussion

The main purpose of this study was to investigate the potential laxative effect of propolis. In the present study, WEP, but not EEP, treatment caused a significant increase in stool weight in normal mice. WEP also ameliorated the clonidine-induced constipation, although had no effect on the loperamide-induced constipation model mice (Figures 1 and 2). Neither WEP nor EEP affected GI transit (Figure 3A). These findings indicate that WEP, but not EEP, had a laxative effect, and indicated that the main constituents found in WEP, but not in EEP, may be responsible for the laxative effect of propolis.

As to the main constituents of WEP and EEP, artepillin C (WEP: 0.59%, EEP: 14%), baccharin (WEP: 0.03%, EEP: 6.8%), and drupanin (WEP: 0.12%, EEP: 1.8%) are relatively little in WEP [28], *p*-coumaric acid (WEP: 3.7%, EEP: 2.5%), 3,4-di-caffeoylqyinic acid (CQA) (WEP: 6.1%, EEP: 3.5%), and 3,5-di-CQA (WEP: 4.9%, EEP: 2.7%) are almost the same amount level in WEP and EEP [28], and chlorogenic acid (WEP: 3.6%, EEP: 0.8%) and other hydrophilic chemical constituents are probably larger in WEP than in EEP. Among other minor chemical constituents, naringenin at 150 mg/kg is reported to have laxative effect in a rat loperamide-induced constipation model [29]. Considering the effective dose of naringenin in [29], micro amount of naringenin in propolis, and the failure of WEP and EEP to have laxative effects in loperamide-induced model in our study, naringenin would not be active constituents of WEP at least in the present study. Taken together, highly hydrophilic compounds including ferulic acid, and isoferulic acid , but not di-caffeoylquinic acids and its metabolites (caffeic acid and quinic acid), might be active constituents of WEP in the present study, however there are no reports showing the laxative effects of these chemical compounds now.

The laxative effect of WEP was seen in the clonidine-induced constipation model, but not in the loperamide-induced model (Figure 2A and B). Clonidine and loperamide are agonists of α_2_ adrenergic receptor and of μ opioid receptor, respectively. The opioid receptor agonists and the α_2_ receptor agonists inhibit endogenous acetylcholine release as results of inhibitions of adenyl cyclase *via* G protein in myenteric plexus, and chronic treatments of opioid receptor agonist or α_2_ adrenergic receptor agonist are reported to increase expressions of G proteins in gastrointestinal tracts in guinea pig [30]. Among opioid agonists, loperamide is classified into piperidine delivatives as well as fentanyl and pethidine. But unlike fentanyl, pethidine, and othe opioid agonists (heroine, morphine, oxycodone, etc.), only loperamide is non-narcotic because it does not reach central nerve system with difficult solubility in water and little absorption into blood flow. Loperamide is estimated to act directly on the intestinal nerve system to induce constipation because loperamide has little central action. On the other hand, clonidine specifically binds to the α_2_ adrenergic receptor of the brainstem [31], and also binds to peripheral adrenergic receptor *via* blood flow to relax intestinal smooth muscle to induce constipation. From these points of view, mechanisms of constipation induced by loperamide and clonidine are analogous to each other, apart from absorption into blood flow and direct interaction to Auerbach’s plexus from gastrointestinal tract. These differences might lead to the result we showed in Figure 3.

Subsequent experiments investigated the direct potency of WEP on the small intestine with the Magnus method (Figure 3B, C, and D). WEP significantly increased the intestinal tension in a dose-dependent manner. The acute response to WEP suggests that WEP has a direct effect on the gastrointestinal tract *in vivo*.

We also evaluated the influence of pre-treatments of GR113808, a 5-HT_4_ receptor antagonist, and atropine, an acetylcholine receptor antagonist on the WEP-induced increase in intestinal tension (Figure 3C and D). No effect was observed for GR113808 on the WEP-induced increase in intestinal tension, while atropine treatment significantly reduced the WEP-induced increase. These findings indicated that the WEP-induced increment of intestinal tension is probably not mediated by the 5-HT_4_ receptor.

In Auerbach's plexus, activated 5-HT_4_ receptor and 5-HT_3_ receptor (M receptor) induce endogenous acetylcholine release from parasympathetic nerve, on the other hand activated 5-HT_2_ receptor (D receptor) directly induces contraction of intestinal smooth muscle. The failure of GR113808 to affect WEP-induce increment of intestinal tension indicates that the acute effect of WEP on intestinal tract was not be mediated by serotonin or serotonin-induced parasympathetic nerve system. From this point of view, WEP may induce the intestinal contraction *via* mechanism of direct stimulation of muscarinic receptor, endogenous acetylcholine release, or parasympathetic nerve stimulation, which is not mediated by serotonin.

The results obtained with the Magnus method demonstrated that WEP increased the tension of ileum immediately after administration (Figure 3B), indicating that unmetabolized ingredients in WEP induced the intestinal smooth muscle contraction, unlike rhein-anthrone which is active metabolite by intestinal flora from sennoside A in *Senna alexandrina*.

Many types of treatment are available for chronic constipation, including dietary fiber, fluids, and exercise. Effective pharmacological agents can be divided into several categories, including “bulk forming agents” (psyllium, bran, etc.), “stool softeners” (docusate sodium/potassium, etc.), “osmotic agents” (polyethylene glycol-lactulose, sorbitol, etc.), “stimulants” (senna, castor oil, bisacodyl, etc.), and “chloride channel activators” (Lubiprostone) [26]. Traditional and herbal medicines are widely used for constipation, and many of these are classified into stimulant types, such as senna (*Senna alexandrina*), aloe (*Aloe barbadensis miller*), or castor oil (*Ricinus communis*). These stimulant-type agents often induce diarrheas as adverse effects at common doses [32,33].

The present study shows that WEP stimulated the ileum isolated from guinea pigs (Figure 3B), on the other hand WEP did not affect gastrointestinal tract in GI transit test (Figure 3A). GI transit test is mainly targeted duodenum and jejunum, meaning not ileum, because charcoal travelled less than 60% of the whole small intestine in any groups in Figure 3A. Though there is no clearly-defined distinction between jejunum and ileum, ileum is more sensitive to enteral toxins [34] and some herbal medicine [35] than jejunum. In our previous study, basal tension of jejunum is 1.5-fold stronger than ileum. The difference between Figure 3A and 3B may attribute to difference of sensitivity between jejunum and ileum.

WEP did not induce an adverse effect of diarrhea in the present experiments (data not shown), unlike senna or castor oil that are reported to induce diarrhea [35]. We hypothesized that this is because of differences in target organs and pharmacological activities. The adverse effect of diarrhea is largely caused by inhibition of water absorption in the large intestine. Among the stimulants, senna (*Senna alexandrina*, rhein anthron) and aloe (*Aloe ferox*, barbaloin) have been identified to stimulate large intestine, which is a water absorptive organ, whereas olive oil and castor oil stimulate small intestine and also inhibits water and nutrition absorption in the small intestine. The data from the present study indicate that WEP does not inhibit absorption and probably act through a different mechanism from that invoked by senna, aloe, or castor oil.

## Conclusions

In conclusion, WEP treatment resulted in laxative effects in normal mice and clonidine-induced constipation mice, but did not induce diarrhea as an adverse side effect. The active ingredient in propolis, which is probably hydrophilic, may therefore stimulate the ileum and increase contractional tension, partly *via* activation of an acetylcholine receptor.

## Abbreviations

WEP: Water extract of propolis; EEP: Ethanol extract of propolis.

## Competing interests

Brazilian green propolis was supplied by API Co., Ltd. (Gifu, Japan). There is no other competing interest.

## Authors' contribution

MK primarily conceived and designed the study, performed the experiments, analyzed the data, and wrote the paper; HI partly performed the experiments, prepared the samples, and participated in the design of the study; KT, YA, MS, and KI participated in the design of the study; HH conceived and organized the study, and participated in the design of the study. All authors read and approved the final manuscript.

## Pre-publication history

The pre-publication history for this paper can be accessed here:

http://www.biomedcentral.com/1472-6882/12/192/prepub
